# A Devastating Diagnosis: Anencephaly With Unexpected Fetal Heartbeat

**DOI:** 10.7759/cureus.67321

**Published:** 2024-08-20

**Authors:** Neil A Nunes, Nishchay W Tekulwar, Ranjit Prasad

**Affiliations:** 1 Department of Radio-Diagnosis, Sree Balaji Medical College and Hospital, Chennai, IND; 2 Radiodiagnosis, Dr Tekulwar Hospital, Allapalli, IND

**Keywords:** gynecology and obstetrics, late diagnosis, general radiology, multiple neural tube defect, anencephaly

## Abstract

This case report describes a severe birth defect called anencephaly in a fetus at 18 weeks of pregnancy. Anencephaly occurs when the upper part of the baby’s brain and skull do not form correctly. This condition is usually detected earlier in pregnancy, often in the first three months. However, this case was discovered at 18 weeks, highlighting the need for careful monitoring throughout pregnancy. The unique aspect of this case is that most pregnancies with anencephaly do not progress this far. This later diagnosis provides important information about the different ways the condition can develop. By studying cases like this, healthcare providers can improve prenatal care and potentially detect such defects earlier, even in the later stages of pregnancy. Overall, this report emphasizes the importance of continuous monitoring during pregnancy and offers insights that could lead to better diagnosis, care, and support for families facing similar situations.

## Introduction

Neural tube defects (NTDs) occur when the neural tube, the structure that eventually becomes the brain and spinal cord, does not fully close during the first month of pregnancy. This incomplete closure can lead to severe birth defects affecting the brain or spine. NTDs affect approximately >1 to 11 out of 1,000 pregnancies [1-3].

There are several different types of NTDs, including myelomeningocele (50%), wherein the spinal cord may be exposed (spina bifida aperta) or covered by a meningeal sac (spina bifida cystica), typically occurring in the thoracolumbar, lumbar, or lumbosacral regions; anencephaly (40%), presenting as a lack of brain and cranial vault; and encephalocele (5%), presenting as a meningeal sac, which may include brain tissue and protrude from the skull, typically occurring in the occipital, parietal, or frontoethmoidal regions [2,3]. Of these, anencephaly is the most severe type of NTD. Babies born with this condition are missing a significant portion of their brain and skull. The severity of anencephaly can vary, from a complete absence of the brain to a partial absence [4]. Its Incidence is around 1:1,000 to 1:20,000 [5]. The occurrence of anencephaly varies significantly across different populations. It is most common in Great Britain and Ireland and least common in Asia, Africa, and South America. Additionally, Caucasian individuals are six times more likely to have anencephaly compared to Black individuals, and females are more frequently affected than males (female to male ratio: 4:1). In the Indian population, the prevalence of anencephaly was reported to be approximately 2.1 per 1,000 births. Anencephaly is more frequently observed in first-time mothers, as well as in both young and older mothers. The condition is believed to be linked to a combination of genetic and environmental factors, indicating a multifactorial origin [6].

Several factors contribute to the risk of NTDs during pregnancy. Women with insulin-dependent diabetes mellitus face a significantly higher risk of major central nervous system malformations, including spina bifida, but strict metabolic control before conception can mitigate this risk. Maternal obesity is another critical factor, with the risk of NTDs increasing as body weight rises, independent of folate intake. Anti-epileptic drugs (AEDs) also pose a danger, as they cross the placenta and disrupt folate metabolism, with the likelihood of congenital anomalies escalating with the number of AEDs used. Hyperthermia, particularly during the first trimester, whether from fever, sauna use, or excessive physical exertion in hot environments, has a strong association with NTDs. Certain medications that interfere with folate metabolism, including antimicrobials, methotrexate, and aspirin, further elevate the risk, making it crucial for women of childbearing age to avoid these drugs when possible. Additionally, excessive intake of preformed vitamin A (>15,000 IU/day) during pregnancy is teratogenic and linked to a higher incidence of NTDs. Smoking during pregnancy, especially in women with the *MTHFR* 677TT genotype, lowers serum folate levels, increasing the risk of NTDs and other birth defects. Although alcohol consumption is known to cause various birth defects, its direct connection to NTDs is less clear, with some evidence suggesting that it may play a role in certain cases [6].

Regarding genetic predisposition, women who have had a fetus affected by anencephaly face an empirical recurrence risk of 3% in any subsequent pregnancy. However, this risk increases to about 10% after conceiving a second fetus with an NTD. This highlights the significance of genetic factors, though environmental influences on NTD development cannot be overlooked. In twin pregnancies, the concordance rate for NTDs is reported to be 7.7% among monozygotic (identical) twins, which is notably higher than the 4.4% rate for dizygotic (fraternal) twins [7-10].

## Case presentation

A 27-year-old rural tribal female, in a non-consanguineous marriage, came to the outpatient department in June 2024 with complaints of amenorrhea since February 2024. She had a regular 28-day menstrual cycle before this. A urine pregnancy test was conducted and confirmed to be positive. There were no previous reports available and no history of prior abortions. The patient had not undergone any antenatal care checkups before this visit, and no previous ultrasonography (USG) reports were available. Additionally, she had not taken any iron or folic acid supplements, and there was no history of any previous medical or surgical conditions. The patient’s general physical examination findings are shown in Table 1.

**Table 1:** General examination of the patient.

Parameter	Value
Blood pressure	128/86 mmHg
Height	152 cm
weight	52 kg
SpO_2_	99%
Pulse	90 beats/minute
Temperature	36.7°C

**Figure 1:**
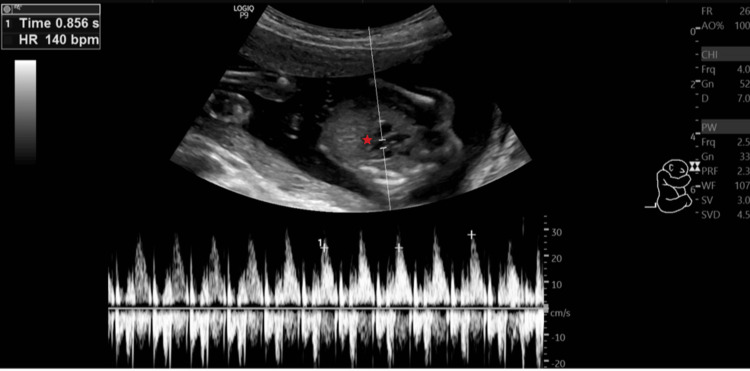
M-mode ultrasonography image demonstrating the fetal heart rate (~140 beats/minute). The star mark (*) shows the position of the fetal heart. The waveform of the fetal heart can be seen at the bottom of the image, and the fetal heart rate can be seen in the top left-hand corner of the image (140 beats/minute).

**Figure 2:**
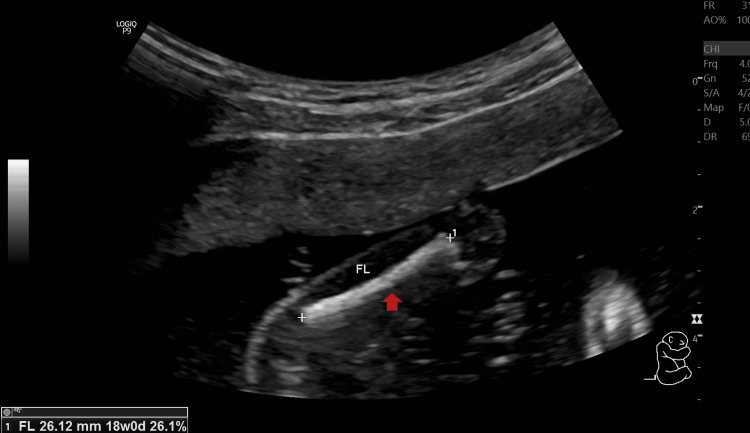
B-mode ultrasonography image showing the femur length of the fetus. The arrow (⬆) points to the femur of the fetus. The measurement of the femur can be read in the bottom left-hand corner of the image. Femur length: 26.12 mm at 18.0 weeks.

**Figure 3:**
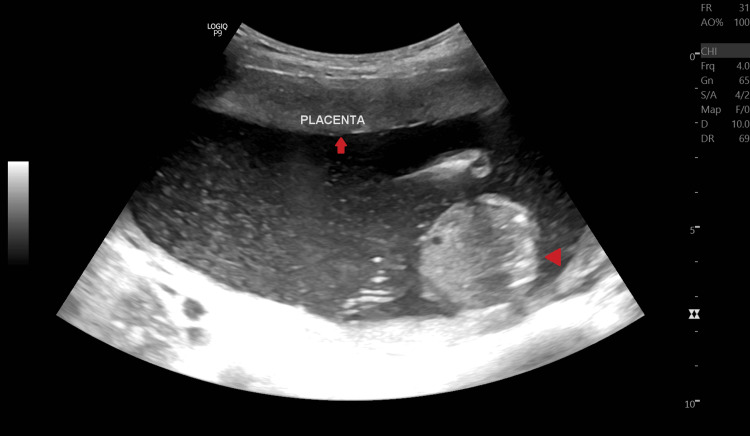
B-mode ultrasonography image showing the position of the placenta on the anterior part of the uterus. The solid arrow (⬆) shows the placenta on the anterior uterine wall, and the arrowhead(◀) shows the fetal abdomen in the axial view.

**Figure 4:**
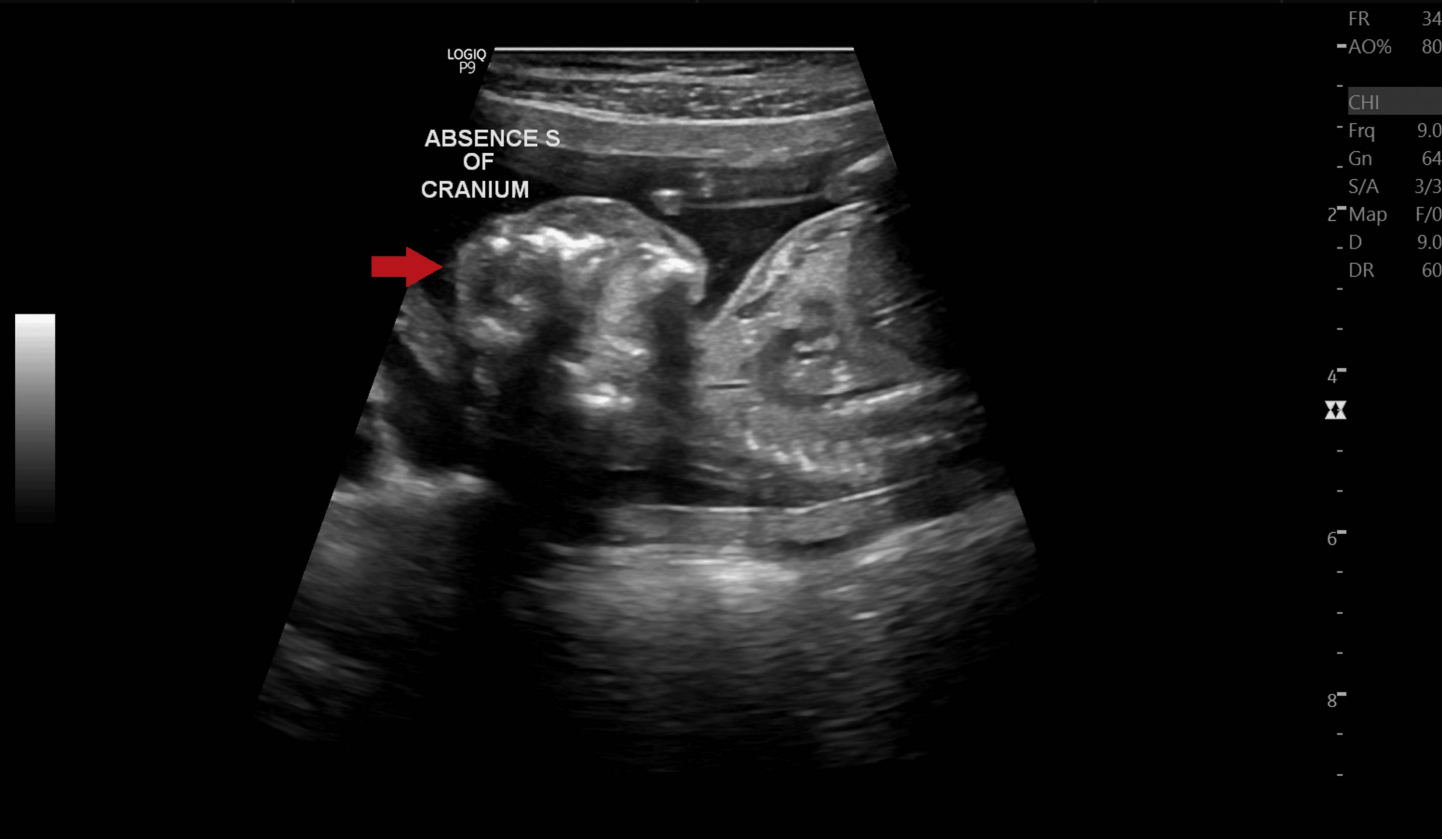
B-mode ultrasonography mid-sagittal image showing the absence of the cranium of the fetus. The arrow (➡) points to the fetal head showing the absence of the cranium.

**Figure 5:**
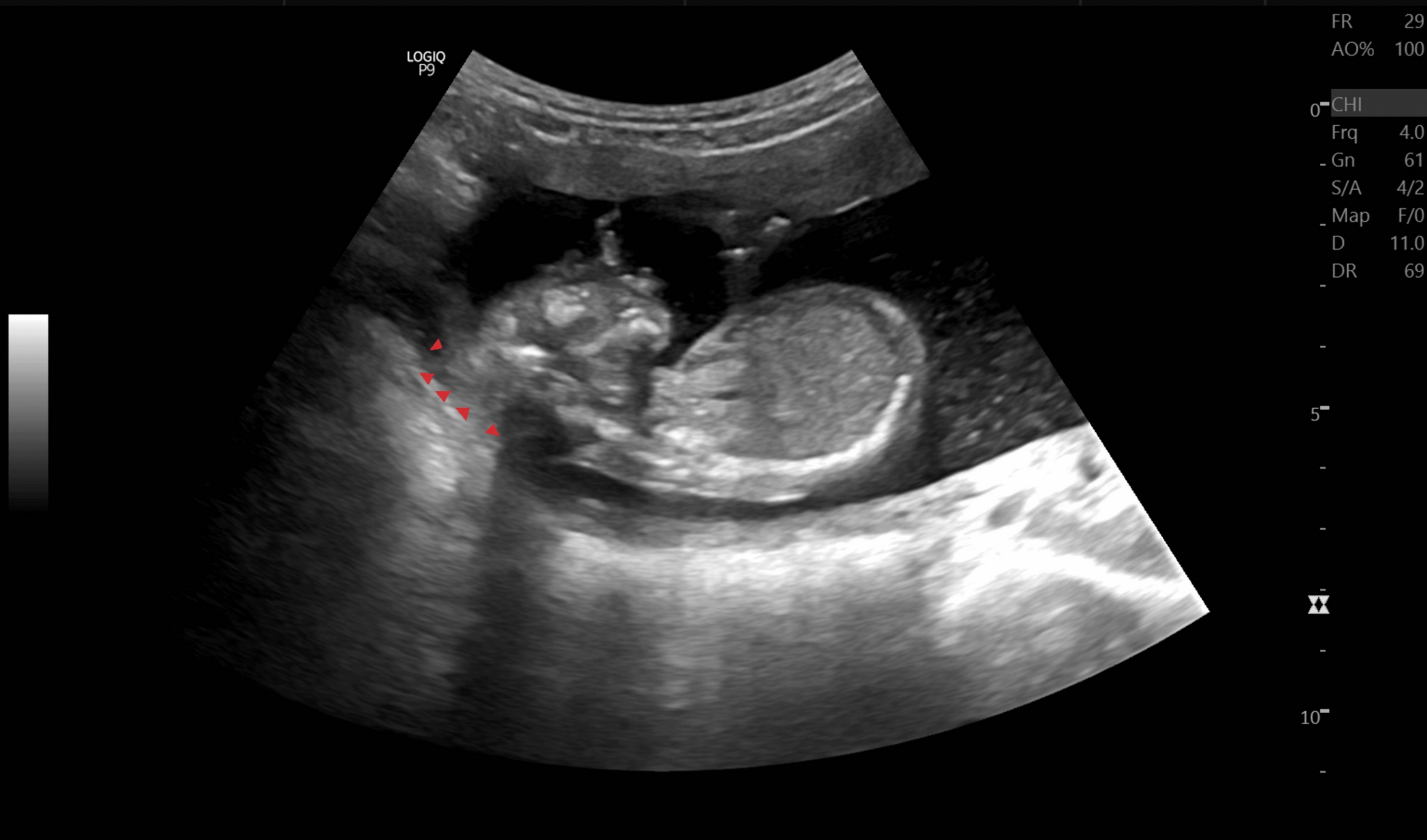
B-mode ultrasonography of the fetus in the mid-sagittal plane. Multiple arrowheads (▶) showing a large amount of angiomatous stroma cephalad to the base of the skull.

On palpation, her fundal height corresponded to 18 weeks of gestation. On USG examination, fetal heart rate was ~140 beats/minute (Figure 1), fetal movements were present, femur length was ~26.1 mm and corresponded to ~18.0 weeks+/- 1 week 4 days (Figure 2), and the amniotic fluid index was ~25.3 cm which suggested polyhydramnios. The placenta was noted to be anterior and not low-lying (Figure 3). The cervical OS appeared to be closed and the cervical length measured ~25.5 mm. A congenital defect was noted in the form of an absence of the skull vault (Figure 4) and a large amount of angiomatous stroma cephalad to the base of the skull (Figure 5), which confirmed the diagnosis of anencephaly.

Subsequently, the patient was counseled about the USG findings, including the diagnosis being incompatible with life. The patient subsequently opted for termination of pregnancy and was referred to an obstetrician for further management. The obstetrician opted for a medical termination of pregnancy. A 25 µg misoprostol tablet was inserted into the posterior fornix of the vagina and was repeated every four hours, with a total dose of 150 µg administered for the induction of labor. This approach was used to achieve cervical ripening. Once the cervix was sufficiently dilated, the delivery was completed with the assistance of forceps.

**Figure 6:**
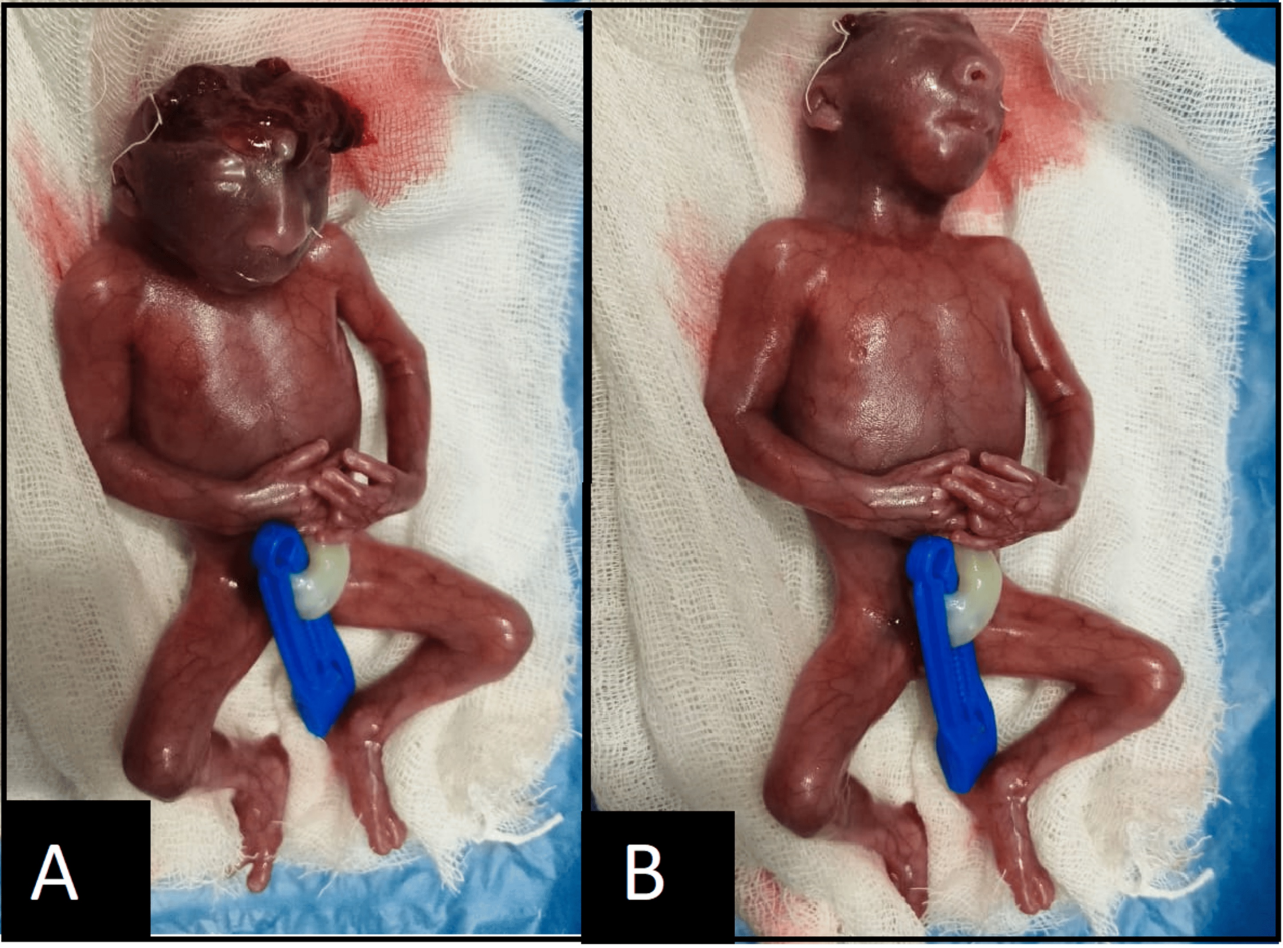
(A) The fetus from above clearly showing the missing cranium. (B) The fetal face. Both images were taken post-delivery.

After the expulsion, the placenta was weighed and was found to be 108 g. The fetus was identified as female, weighed 220 g, and showed gross deformity with an absent cranium and exposed brain tissue (Figure 6). The mother’s post-procedural stay was uneventful.

## Discussion

In recent years, widespread screening and testing and government preventive programs have reduced the number of newborns born with major NTDs including anencephaly. However, compliance with the programs remains an issue [1].

Folic acid deficiency is a primary cause of NTD. Consuming folic acid supplements early in pregnancy can significantly reduce the risk of this severe birth defect. This is especially important for women in developing countries and those with limited financial resources, as these populations are at a higher risk [1-5]. Anencephaly is often accompanied by other health issues. These can include additional NTDs such as spina bifida, particularly affecting the cervical spine. Moreover, babies with anencephaly may have heart defects, cleft lip or palate, diaphragm abnormalities, spinal abnormalities beyond the brain, skeletal issues such as clubfoot, digestive problems such as omphalocele, and urinary tract complications, with hydronephrosis being the most common.

It is also important to note that in our case the patient had not initiated iron and folic acid tablets before or during the first trimester, although they are available through government schemes free of cost. This was probably due to the patient being of an illiterate tribal background and having little to no formal education and thus was not aware of such schemes.

Anencephaly is strongly associated with significantly elevated levels of alpha-fetoprotein in the mother’s blood, often exceeding normal levels by two and a half times. In fact, anencephaly is the NTD most likely to cause such a dramatic increase. [7]. In our case, this test was not conducted due to the poor financial status of the patient.

USG examinations can detect anencephaly as early as the 11th week of pregnancy. This imaging method is highly accurate, non-invasive, and cost-effective for identifying this condition. Key USG findings include the absence of brain tissue and skull above the eye sockets, sometimes with remnants of the occipital bone or midbrain. In less severe cases, known as exencephaly, some brain tissue may be present. Other USG signs of anencephaly include reduced fetal size, an abnormal shape of the head resembling a frog’s eye or Mickey Mouse ears, and excessive amniotic fluid due to difficulty swallowing [8,9].

Babies born with anencephaly cannot survive. While there is a small chance of around 2.5% of having another child with an NTD, consuming folic acid before and during pregnancy can significantly reduce this risk [1-3,8,11].

MRI can help distinguish anencephaly from other similar brain abnormalities. These include conditions such as cranium bifidum occultum (open fontanels without brain or membrane protrusion), acrania (absence of the skull), exencephaly (exposed brain outside the skull), meningoencephalocele (brain and membrane protrusion), atelencephalic microcephaly (abnormally small brain with specific developmental issues), and hydranencephaly (destroyed brain tissue replaced by fluid) [12]. Accurately differentiating between these conditions is essential as some, such as meningoencephalocele, may allow for potential surgical repair and survival [12].

## Conclusions

Anencephaly, a devastating NTD, underscores the critical importance of early and regular antenatal care. This case highlights the unique and tragic consequences of this condition that sometimes even though there may be a heartbeat, it may not be possible for the fetus to survive. It also emphasizes the need for comprehensive prenatal screening. Preventive measures, particularly folic acid supplementation, are instrumental in reducing the incidence of anencephaly. Adequate nutrition, including a diet rich in folic acid, plays a pivotal role in fetal development. It is imperative to educate women of childbearing age about the significance of folic acid intake before and during pregnancy. Early detection through USG examinations can provide essential information for parents and healthcare providers to make informed decisions. Termination of the pregnancy may sometimes be the most logical and the best choice keeping in mind the health of the patient, but sometimes due to cultural and religious reasons, it may not always be chosen. Moreover, this choice also carries a significant emotional toll on the patient. While advancements in medical science continue to evolve, prevention remains the cornerstone in combating this tragic condition. By prioritizing antenatal care, promoting folic acid supplementation, and raising awareness about the importance of maternal health, we can strive to reduce the occurrence of anencephaly and improve maternal and child health outcomes.
